# Prevalence of and factors associated with renal dysfunction in rheumatoid arthritis patients: a cross-sectional study in community hospitals

**DOI:** 10.1007/s10067-017-3804-5

**Published:** 2017-09-07

**Authors:** Shunsuke Mori, Tamami Yoshitama, Naoyuki Hirakata, Yukitaka Ueki

**Affiliations:** 1 Department of Rheumatology, Clinical Research Center for Rheumatic Diseases, NHO Kumamoto Saishunsou National Hospital, 2659 Suya, Kohshi, Kumamoto 861-1196 Japan; 2 Yoshitama Clinic for Rheumatic Diseases, Kirishima, Kagoshima 899-5117 Japan; 3 Rheumatic and Collagen Disease Center, Sasebo Chuo Hospital, Sasebo, Nagasaki 857-1195 Japan

**Keywords:** Glomerular filtration rate, Prevalence, Renal dysfunction, Rheumatoid arthritis, Risk factors

## Abstract

This study was designed to determine the prevalence of renal dysfunction in rheumatoid arthritis (RA) patients and to identify factors associated with this complication. Between October 2014 and May 2015, we consecutively recruited RA patients at rheumatology sections of community hospitals in Japan. Each patient’s absolute and body surface area (BSA)-indexed estimated glomerular filtration rate (eGFR) values were measured twice over a 3-month interval. Renal dysfunction was defined as absolute eGFR or BSA-indexed eGFR < 60. Albuminuria and hematuria were also recorded. Associations between renal dysfunction and possible risk factors were examined by multivariate logistic regression analyses. A total of 1908 outpatients with RA were included in this study. The prevalence of renal dysfunction based on absolute eGFR and BSA-indexed eGFR was 33.8 and 18.6%, respectively. Albuminuria was observed in 8.1% of this patient cohort, and the prevalence of hematuria was 7.5%. Advanced age (odds ratio [OR] 7.24, *p* < 0.001), female sex (OR 3.12, *p* < 0.001), hypertension (OR 2.22, *p* < 0.001), and obesity (OR 0.59, *p* < 0.001) were independently associated with the risk of absolute eGFR-based renal dysfunction. Advanced age (OR 5.19, *p* < 0.001) and hypertension (OR 3.05, *p* < 0.001) also had associations with BSA-indexed eGFR-based renal dysfunction. RA duration, stages, severity, and cumulative steroid dose were considered significant risk factors in univariate analyses, but their associations were less potent after adjustment for other covariates. Renal dysfunction is relatively common in RA patients and is mainly associated with advanced age and hypertension but not with RA-related factors.

## Introduction

Rheumatoid arthritis (RA) is an autoimmune disease in which persistent inflammatory changes occur in multiple synovial joints and many extra-articular organs. Renal involvement is one of the most important complications in the management of RA patients because of its prognostic value and therapeutic implications [1, 2]. Although it has been reported that a considerable number of RA patients also have proteinuria, hematuria, and renal dysfunction, the figures reported for the prevalence and incidence of renal involvement vary depending on the criteria used to define the disease and the patient populations examined [1, 3–8].

The factors associated with renal dysfunction in RA patients remain controversial. Previous studies of renal biopsy findings and clinicopathologic correlations showed that the most predominant pathological findings in clinical renal disease of RA patients were secondary amyloidosis, membranous nephropathy related to the use of antirheumatic drugs, mesangial glomerulonephritis possibly related to RA itself, and rheumatoid vasculitis commonly associated with rapidly progressive glomerulonephritis [9–11]. These studies were performed with RA patients who had received old antirheumatic drugs, such as gold, penicillamine, and bucillamine, which are known to be causative drugs for membranous nephropathy. These nephrotoxic drugs are, however, seldom used in current RA treatment. Secondary amyloidosis is now relatively rare in RA because of advances in the management and control of RA activity [12]. Although the contributory role of inflammation on renal dysfunction and renal damage was reported in previous studies on the general population [13–15], inconsistent results were obtained from recent studies on RA patients [5–8, 16–18].

The current scope of renal involvement in RA patients may widen. In daily practice, we encounter cases in which RA and renal disease coincide more often than we did in the past, despite the popularity of novel antirheumatic drugs, including biological agents, which can more effectively suppress the systemic inflammation associated with RA. The markedly increased risk of developing cardiovascular disease (CVD) has been recognized in RA patients for many years [19, 20], and this may contribute to the increased risk of premature death of this patient population [21–23]. Several studies have suggested that reduced renal function may be associated with CVD risk in RA patients [16, 17, 24, 25]. Appropriate risk management for impaired renal function is therefore important in reducing the risk of CVD mortality and improving outcomes of RA patients.

To determine the prevalence of renal dysfunction in RA patients, we performed a cross-sectional study at rheumatology sections of community hospitals in Japan. Each patient’s absolute and body surface area (BSA)-indexed estimated glomerular filtration rate (eGFR) values were measured twice over a 3-month interval, and average values were then used to assess renal function. We also evaluated the independent associations of renal dysfunction with demographic and RA-related characteristics, the presence of traditional cardiovascular risk factors, and the use of RA medications.

## Patients and methods

### Patients and study design

Between October 2014 and May 2015, we consecutively recruited patients with RA from outpatient clinics for rheumatic diseases at the following community hospitals in Japan: NHO Kumamoto Saishunsou National Hospital, Yoshitama Clinic for Rheumatic Diseases, and Sasebo Chuo Hospital. Each patient was included once. Regardless of the reasons for their visits, eligible patients (namely, those who fulfilled the 1987 American College of Rheumatology [ACR] criteria or the 2010 ACR/European League Against Rheumatism [EULAR] criteria for diagnosis of RA) were enrolled in this study, unless they met any of the following exclusion criteria: being under 18 years of age, having acute kidney disease, receiving renal replacement therapy, or being pregnant.

For each patient, demographic characteristics, RA-related factors such as RA duration, Steinbrocker stage, clinical disease activity index (CDAI), health assessment questionnaire (HAQ), positivity of anti-cyclic citrullinated peptide antibodies (anti-CCP Abs), levels of serum C-reactive protein (CRP), and the current use of disease-modifying antirheumatic drugs (DMARDs), nonsteroidal anti-inflammatory drugs (NSAIDs), and steroids, together with traditional cardiovascular risk factors including hypertension, diabetes mellitus, smoking, body mass index (BMI), and levels of serum low-density lipoprotein cholesterol (LDL-C), were examined and recorded at inclusion. Serum levels of creatinine, together with the presence of albuminuria and hematuria, were also examined and recorded at the same time. Serum creatinine was measured twice over a 3-month interval for individual patients.

Hypertension was defined as systolic blood pressure of 140 mmHg or greater, diastolic blood pressure of 90 mmHg or greater, or the use of antihypertensive medications. Diabetes was defined as fasting blood glucose levels of 126 mg/dl or higher, serum HbAc1 higher than 6.0%, or the use of diabetes medications. Hematuria was defined as the presence of five or more red blood cells per high-power field in three of three consecutive centrifuged specimens obtained at least 1 week apart. Urinary albumin was determined by a semi-quantitative urine dipstick testing kit (BM test MAU II, Roche Diagnostics K.K., Tokyo, Japan). Albuminuria was defined as “2+” or more in dipstick testing, which corresponds to 50 mg/l or higher of urinary albumin.

NSAID intake was calculated according to the Assessment of Spondyloarthritis International Society (ASAS)-NSAID scoring system [26]. Information on the name and daily dose of NSAIDs as well as the number of days with NSAID intake during the previous 3 months was collected. To express results as a mean daily intake over the previous 3 months, the following formula was used: ASAS-NSAID score = (NSAID equivalent score) × (days of intake during period of interest) × (intake days per week) / (period of interest in days) [26]. Cumulative doses of methotrexate (MTX) and steroids (prednisolone equivalent) prescribed after initiation of RA treatment in the participating hospitals were also calculated for each user of these drugs. Information on weekly MTX dose, daily steroid dose, and duration of treatment was collected by reviewing patients’ medical records.

### Renal function

An eGFR was first calculated according to the following equation, which were developed for Japanese patients and officially approved by the Japanese Society of Nephrology: eGFR (ml/min/1.73 m^2^) = 194 × (serum creatinine [mg/dl])^−1.094^ × (age)^−0.287^ × 0.739 (if female) [27]. A value calculated by this formula is an indexed eGFR that is adjusted to a standard BSA of 1.73 m^2^, but this indexation of eGFR can be misleading in populations with unusual anthropometric data, such as those containing obese or lean persons [28, 29]. In the present study, the mean BSA value of participants was quite different from the standard BSA: it was 1.52 m^2^. An overestimation of eGFR may result if we use BSA-indexed values. Thus, we studied the prevalence and possible risk factors of renal dysfunction using both absolute eGFR values and BSA-indexed eGFR values. The above-mentioned calculated value (BSA-indexed eGFR) was converted, using each patient’s BSA, to an unindexed eGFR value (absolute eGFR): absolute eGFR = indexed eGFR × patient’s BSA / 1.73 m^2^ [30]. The BAS was calculated using the Du Bois formula: BSA (m^2^) = 0.007184 × (weight [kg])^0.425^ × (height [cm])^0.725^. Two measurements of eGFR were taken over a 3-month interval for individual patients, and average values were used in this study. Renal dysfunction was defined as absolute eGFR < 60 ml/min or BSA-indexed eGFR < 60 ml/min/1.73 m^2^. eGFR stages were determined according to the Kidney Disease Improving Global Outcomes (KDIGO 2012) guidelines [31].

### Statistical analysis

For comparisons of categorical variables between the groups with and without renal dysfunction, a statistical analysis was performed using the chi-square test. Continuous variables were assessed by the independent-measures *t* test or the Mann-Whitney *U* test. Multivariate logistic regression analysis was performed to evaluate the association between renal dysfunction as a dependent variable and a set of independent variables considered to be significant risk factors in univariate analyses. We also included, as independent variables, factors that were not significant in univariate analyses but could be confounding or clinically relevant variables. A backward stepwise selection procedure was used to select significant independent variables. The strength of association between renal dysfunction and these independent variables was estimated using odds ratios (ORs) and 95% confidence intervals (95% CIs). In addition, the receiver operating characteristic (ROC) curves and the corresponding area under the curve (AUC) values were calculated to provide an index of validity of multivariate logistic regression models.

For all tests, probability values (*p* values) < 0.05 were considered to indicate statistical significance. All calculations were performed using PASW Statistics version 22 (SPSS Japan Inc., Tokyo, Japan).

## Results

### Prevalence of renal dysfunction

A total of 1908 patients with RA (350 men and 1558 women) were prospectively included in this study. Among these patients, 645 (33.8%) were found to have renal dysfunction, defined as absolute eGFR < 60 ml/min (Table 1). The prevalence of renal dysfunction increased with age, affecting more than 50% of people over the age of 70 years, and it was higher in women (female 36.6% versus male 21.1%). These trends were observed in all eGFR stages of renal dysfunction. Only a small percentage of patients (2.1%) had severe or end-stage kidney disease defined as absolute eGFR < 30 ml/min. The prevalence of albuminuria was 8.1% (155 out of 1908 patients), and among these patients, 58.7% were categorized as having renal dysfunction (Table 2). The prevalence of albuminuria was 5.1% (64 out of 1263 patients) in the group without renal dysfunction and 14.1% (91 out of 645 patients) in the renal dysfunction group. The prevalence of hematuria was 7.5% (143 out of 1908 patients), and among these patients, 48.3% had renal dysfunction. The prevalence of hematuria was 5.9% (74 out of 1263) in the group without renal dysfunction and 10.7% (69 out of 645 patients) in the renal dysfunction group.

**Table 1:** Prevalence of renal dysfunction in RA patients after stratification according to age or sex (*n* = 1908)

	Total (*n* = 1908)	Age (years)					Female/male	
		< 50 (*n* = 281)	≥ 50 and < 60 (*n* = 378)	≥ 60 and < 70 (*n* = 611)	≥ 70 and < 80 (*n* = 442)	≥ 80 (*n* = 196)	Female (*n* = 1558)	Male (*n* = 350)
Absolute eGFR,^a^ mean (SD)	69.0 (20.7)	88.3 (18.7)	76.9 (17.7)	69.9 (16.3)	58.1 (16.6)	47.4 (14.8)	67.5 (20.2)	75.2 (21.5)
eGFR ranges, patient number (%)								
≥ 60	1263 (66.2)	268 (95.4)	320 (84.7)	450 (73.6)	189 (42.8)	36 (18.4)	987 (63.4)	276 (78.9)
< 60	645 (33.8)	13 (4.6)	58 (15.3)	161 (26.4)	253 (57.2)	160 (81.6)	571 (36.6)	74 (21.2)
≥ 45 and < 60	412 (21.6)	11 (3.9)	47 (12.4)	128 (20.9)	158 (35.7)	68 (34.7)	360 (23.1)	52 (14.9)
≥ 30 and < 45	193 (10.1)	2 (0.7)	8 (2.1)	30 (4.9)	82 (18.6)	71 (36.2)	176 (11.3)	17 (4.9)
≥ 15 and < 30	37 (1.9)	0	3 (0.8)	3 (0.5)	12 (2.7)	19 (9.7)	33 (2.1)	4 (1.1)
< 15	3 (0.2)	0	0	0	1 (0.2)	2 (1.0)	2 (0.1)	1 (0.3)
BSA-indexed eGFR,^a^ mean (SD)	78.4 (21.8)	96.4 (19.3)	84.9 (19.4)	79.7 (18.5)	68.1 (18.9)	58.8 (17.6)	78.8 (22.1)	76.4 (20.2)
eGFR ranges, patient number (%)								
≥ 60	1553 (81.4)	276 (98.2)	347 (91.8)	538 (88.1)	297 (67.2)	95 (48.5)	1263 (81.1)	290 (82.9)
< 60	355 (18.6)	5 (1.8)	31 (8.2)	73 (11.9)	145 (32.8)	101 (51.5)	295 (18.9)	60 (17.1)
≥ 45 and < 60	263 (13.8)	4 (1.4)	25 (6.6)	61 (10.0)	110 (24.9)	63 (32.1)	217 (13.9)	46 (13.1)
≥ 30 and < 45	74 (3.9)	1 (0.4)	4 (1.1)	9 (1.5)	32 (7.2)	28 (14.3)	63 (4.0)	11 (3.1)
≥ 15 and < 30	15 (0.8)	0	2 (0.5)	3 (0.5)	2 (0.5)	8 (4.1)	13 (0.8)	2 (0.6)
< 15	3 (0.2)	0	0	0	1 (0.2)	2 (1.0)	2 (0.1)	1 (0.3)

*RA* rheumatoid arthritis, *eGFR* estimated glomerular filtration rate, *BSA* body surface area, *SD* standard deviation

^a^BSA-indexed eGFR was first calculated using the following equation: BSA-indexed eGFR (ml/min/1.73 m^2^) = 194 × (serum creatinine [mg/dl])^−1.094^ × (age)^−0.287^ × 0.739 (if female) and was then converted to an unindexed eGFR (an absolute eGFR) using each patient’s BSA. Absolute eGFR (ml/min) = eGFR × BSA / 1.73 m^2^. Two measurements of eGFR were taken over a 3-month interval for individual patients, and average values were used

**Table 2:** Prevalence of renal dysfunction in RA patients after stratification according to the presence of albuminuria or hematuria (*n* = 1908)

	Total (*n* = 1908)	Urine albumin		Hematuria	
		< “2+” (*n* = 1753)	≥ “2+” (*n* = 155)	Negative (*n* = 1765)	Positive (*n* = 143)
Absolute eGFR,^a^ mean (SD)	69.0 (20.7)	70.0 (20.3)	57.5 (21.8)	69.6 (20.7)	60.7 (17.9)
eGFR ranges, patient number (%)					
≥ 60	1263 (66.2)	1199 (68.4)	64 (41.3)	1189 (67.4)	74 (51.7)
< 60	645 (33.8)	554 (31.6)	91 (58.7)	576 (32.6)	69 (48.3)
≥ 45 and < 60	412 (21.6)	375 (21.4)	37 (23.9)	372 (21.1)	40 (28.0)
≥ 30 and < 45	193 (10.1)	149 (8.5)	45 (29.0)	170 (9.6)	23 (16.1)
≥ 15 and < 30	37 (1.9)	29 (1.7)	7 (4.5)	32 (1.8)	5 (3.5)
< 15	3 (0.2)	1 (0.1)	2 (1.3)	2 (0.1)	1 (0.7)
BSA-indexed eGFR,^a^ mean (SD)	78.4 (21.8)	79.5 (21.3)	65.5 (22.4)	79.0 (21.8)	70.2 (19.3)
eGFR ranges, patient number (%)					
≥ 60	1553 (81.4)	1468 (83.7)	85 (54.8)	1449 (82.1)	104 (72.7)
< 60	355 (18.6)	285 (16.3)	70 (45.2)	316 (17.9)	39 (27.3)
≥ 45 and < 60	263 (13.8)	217 (12.4)	46 (29.7)	238 (13.5)	25 (17.5)
≥ 30 and < 45	74 (3.9)	57 (3.3)	17 (11.0)	63 (3.6)	11 (7.7)
≥ 15 and < 30	15 (0.8)	10 (0.6)	5 (3.2)	13 (0.7)	2 (1.4)
< 15	3 (0.2)	1 (0.1)	2 (1.3)	2 (0.1)	1 (0.7)

*RA* rheumatoid arthritis, *eGFR* estimated glomerular filtration rate, *BSA* body surface area, *SD* standard deviation

^a^BSA-indexed eGFR was first calculated using the following equation: BSA-indexed eGFR (ml/min/1.73 m^2^) = 194 × (serum creatinine [mg/dl])^−1.094^ × (age)^−0.287^ × 0.739 (if female) and was then converted to an unindexed eGFR (an absolute eGFR) using each patient’s BSA. Absolute eGFR (ml/min) = eGFR × BSA / 1.73 m^2^. Two measurements of eGFR were taken over a 3-month interval for individual patients, and average values were used

When renal dysfunction was defined as BSA-indexed eGFR < 60 min/ml/1.73 m^2^, its prevalence was 18.6% (Table 1). Like the data obtained based on absolute eGFR values, the prevalence of BSA-indexed eGFR-based renal dysfunction was markedly higher in elderly patients, yet there was no significant difference between men and women. Similar results were observed in all eGFR stages of renal dysfunction. The rate of patients with severe or end-stage kidney disease defined as BSA-indexed eGFR < 30 ml/min/1.73 m^2^ was 1.0%. Among patients with albuminuria, 45.2% had renal dysfunction (Table 2). The prevalence of albuminuria was 5.5% (85 out of 1553 patients) in the group without renal dysfunction and 19.7% (39 out of 355 patients) in the renal dysfunction group. Among patients with hematuria, 27.3% had renal dysfunction. The prevalence of hematuria was 6.7% (104 out of 1553 patients) in the group without renal dysfunction and 11.0% (39 out of 355 patients) in the renal dysfunction group.

### Comparisons of demographic and clinical characteristics between RA patients with and without renal dysfunction based on absolute eGFR values

Patients with renal dysfunction defined as absolute eGFR < 60 ml/min were significantly older (72.0 versus 58.8 years, *p* < 0.001) and had longer RA duration (12.0 versus 10.1 years, *p* < 0.001) (Table 3). This patient group was more often diagnosed as being at Steinbrocker classification stages III and IV (52.7 versus 45.2%, *p* = 0.002), and had a significantly higher mean level of HAQ (0.56 versus 0.35, *p* < 0.001), but there was no significant difference in other RA-related indexes such as CDAI and anti-CCP positivity between the two patient groups. Renal dysfunction was significantly associated with female sex (88.5 versus 78.1%, *p* < 0.001), but obesity (BMI > 25 kg/m^2^) was less common in renal dysfunction patients (16.6 versus 23.8%, *p* < 0.001). Hypertension and diabetes mellitus were more frequently observed in the renal dysfunction group (49.0 versus 24.4%, *p* < 0.001; 12.7 versus 8.9%, *p* = 0.010, respectively), but there was no significant difference in mean serum LDL-C concentrations. The rate of current or ex-smokers was lower in the renal dysfunction group (14.9 versus 24.2%, *p* < 0.001).

**Table 3:** Demographic, clinical, and laboratory characteristics of RA patients with and without renal dysfunction based on absolute eGFR values

	Total (*n* = 1908)	With renal dysfunction^a^ (*n* = 645)	Without renal dysfunction^a^ (*n* = 1263)	*p* value^b^
Age, years, mean (SD)	63.3 (13.0)	72.0 (9.6)	58.8 (12.3)	< 0.001
≥ 65 years, patient number (%)	946 (49.6)	513 (79.5)	433 (34.3)	< 0.001
Male/female	350/1558	74/571	276/987	< 0.001
RA duration, years, mean (SD)	10.8 (9.5)	12.0 (10.7)	10.1 (8.8)	< 0.001
≤ 3 years, patient number (%)	400 (21.0)	114 (17.7)	286 (22.6)	0.012
Anti-CCP (+), patient number (%)	1486 (77.9)	503 (78.0)	983 (77.8)	0.95
Stage III/IV, patient number (%)	911 (47.7)	340 (52.7)	571 (45.2)	0.002
CDAI, mean (SD)	6.2 (6.7)	6.3 (6.4)	6.2 (6.8)	0.91
HAQ, mean (SD)	0.42 (0.65)	0.56 (0.77)	0.35 (0.56)	< 0.001
≥ 1.0, patient number (%)	331 (17.3)	156 (24.2)	175 (13.9)	< 0.001
< 0.25, patient number (%)	671 (35.2)	278 (43.1)	393 (31.1)	< 0.001
Serum CRP, mg/dl, mean (SD)	0.36 (0.93)	0.42 (1.13)	0.33 (0.80)	0.050
BMI, kg/m^2^, mean (SD)	22.5 (3.7)	22.1 (3.2)	22.8 (3.9)	< 0.001
> 25, patient number (%)	407 (21.3)	107 (16.6)	300 (23.8)	< 0.001
< 18.5, patient number (%)	196 (10.3)	70 (10.9)	126 (10.0)	0.58
Hypertension, patient number (%)	624 (32.7)	316 (49.0)	308 (24.4)	< 0.001
NIDDM, patient number (%)	194 (10.2)	82 (12.7)	112 (8.9)	0.010
Serum LDL-C, mg/dl, mean (SD)	111.4 (28.8)	110.7 (29.7)	111.8 (28.4)	0.45
≥ 140 mg/dl, patient number (%)	274 (14.4)	103 (16.0)	171 (13.5)	0.17
Current/ex-smokers, number (%)	402 (21.1)	96 (14.9)	306 (24.2)	< 0.001
Use of biologics, patient number (%)	641 (33.6)	239 (37.1)	402 (31.8)	0.024
MTX use, patient number (%)	985 (51.6)	281 (43.6)	704 (55.7)	< 0.001
Dose, mg/week, mean (SD)	7.9 (3.5)	6.2 (3.0)	8.2 (3.5)	< 0.001
Cumulative dose, g, median (IQR)	2.8 (1.2–4.7)	2.8 (1.2–4.4)	2.7 (1.2–4.8)	0.19
NSAID use, patient number (%)	488 (25.6)	156 (24.2)	332 (26.3)	0.35
ASAS-NSAID score, mean (SD)	48.5 (21.0)	49.4 (20.0)	48.1 (21.5)	0.52
Concurrent steroids, number (%)	237 (12.4)	81 (12.6)	156 (12.4)	0.88
Steroid use, patient number (%)	585 (30.7)	199 (30.9)	386 (30.6)	0.92
Dose, mg/day, mean (SD)	3.0 (2.1)	2.9 (1.8)	3.0 (2.2)	0.49
Cumulative dose, g, median (IQR)	3.5 (0.9–10.9)	5.0 (1.5–14.6)	3.1 (0.7–9.3)	< 0.001

*RA* rheumatoid arthritis, *eGFR* estimated glomerular filtration rate, *anti-CCP* anti-citrullinated peptide antibodies, *CDAI* clinical disease activity index, *HAQ* health assessment questionnaire, *CRP* C-reactive protein, *BMI* body mass index, *NIDDM* non-insulin-dependent diabetes mellitus, *LDL-C* low-density lipoprotein cholesterol, *MTX* methotrexate, *NSAIDs* nonsteroidal anti-inflammatory drugs, *ASAS* Assessment of Spondyloarthritis International Society, *SD* standard deviation, *IQR* interquartile range

^a^Defined as an absolute eGFR < 60 ml/min. Average values of two measurements of eGFR taken over a 3-month interval were used

^b^Comparison between RA patients with renal dysfunction and those without this complication

### Comparisons of demographic and clinical characteristics between RA patients with and without renal dysfunction based on BSA-indexed eGFR values

Renal dysfunction defined as BSA-indexed eGFR < 60 ml/min/1.73 m^2^ was significantly associated with age (73.1 versus 61.0 years, *p* < 0.001) but not with sex (Table 4). RA duration was significantly longer in the renal dysfunction group (12.7 versus 10.3 years, *p* < 0.001), and this patient group showed a higher mean level of HAQ (0.59 versus 0.38, *p* < 0.001). Anti-CCP positive rates were similar between the two patient groups. Hypertension and diabetes mellitus were more frequently present in the renal dysfunction group (60.1 versus 26.3%, *p* < 0.001; 17.2 versus 8.6%, *p* < 0.001, respectively), but there was no significant association of renal dysfunction with obesity (BMI > 25 kg/m^2^), smoking history, or serum LDL-C levels.

**Table 4:** Demographic, clinical, and laboratory characteristics of RA patients with and without renal dysfunction based on BSA-indexed eGFR values

	With renal dysfunction^a^ (*n* = 355)	Without renal dysfunction^a^ (*n* = 1553)	*p* value^b^
Age, years, mean (SD)	73.1 (9.6)	61.0 (12.7)	< 0.001
≥ 65 years, patient number (%)	294 (82.8)	652 (42.0)	< 0.001
Male/female	60/295	290/1263	0.49
RA duration, years, mean (SD)	12.7 (11.2)	10.3 (9.0)	< 0.001
≤ 3 years, patient number (%)	55 (15.5)	345 (22.2)	0.005
Anti-CCP (+), patient number (%)	280 (78.9)	1206 (77.7)	0.67
Stage III/IV, patient number (%)	187 (52.7)	724 (46.6)	0.045
CDAI, mean (SD)	6.3 (6.7)	6.2 (6.6)	0.84
HAQ, mean (SD)	0.59 (0.79)	0.38 (0.60)	< 0.001
≥ 1.0, patient number (%)	87 (24.5)	244 (15.7)	< 0.001
< 0.25, patient number (%)	157 (44.2)	514 (33.1)	< 0.001
Serum CRP, mg/dl, mean (SD)	0.47 (1.31)	0.33 (0.81)	0.014
BMI, kg/m^2^, mean (SD)	22.7 (3.4)	22.5 (3.7)	0.31
> 25, patient number (%)	79 (22.3)	328 (21.1)	0.67
< 18.5, patient number (%)	29 (8.2)	167 (10.8)	0.18
Hypertension, patient number (%)	216 (60.1)	408 (26.3)	< 0.001
NIDDM, patient number (%)	61 (17.2)	133 (8.6)	< 0.001
Serum LDL-C, mg/dl, mean (SD)	112.4 (31.3)	111.2 (28.2)	0.50
≥ 140 mg/dl, patient number (%)	61 (17.4)	213 (13.6)	0.094
Current/ex-smokers, number (%)	64 (18.0)	338 (21.8)	0.13
Use of biologics, patient number (%)	135 (38.0)	506 (32.6)	0.054
MTX use, patient number (%)	141 (39.4)	844 (54.3)	< 0.001
Dose, mg/week, mean (SD)	6.7 (3.0)	8.4 (3.6)	< 0.001
Cumulative dose, g, median (IQR)	2.7 (1.3–4.3)	2.8 (1.2–4.7)	0.22
NSAID use, patient number (%)	91 (25.6)	397 (25.6)	1.00
ASAS-NSAID score, mean (SD)	49.8 (19.9)	48.2 (21.2)	0.50
Concurrent steroids, number (%)	54 (15.2)	183 (11.8)	0.094
Steroid use, patient number (%)	127 (35.8)	458 (29.5)	0.022
Dose, mg/day, mean (SD)	3.0 (1.9)	2.9 (2.1)	0.80
Cumulative dose, g, median (IQR)	5.0 (1.5–14.6)	3.2 (0.8–9.5)	< 0.001

*RA* rheumatoid arthritis, *eGFR* estimated glomerular filtration rate, *anti-CCP* anti-citrullinated peptide antibodies, *CDAI* clinical disease activity index, *HAQ* health assessment questionnaire, *CRP* C-reactive protein, *BMI* body mass index, *NIDDM* non-insulin-dependent diabetes mellitus, *LDL-C* low-density lipoprotein cholesterol, *MTX* methotrexate, *NSAIDs* nonsteroidal anti-inflammatory drugs, *ASAS* Assessment of Spondyloarthritis International Society, *SD* standard deviation, *IQR* interquartile range

^a^Defined as a BSA-indexed eGFR <60 ml/min/1.73 m^2^. Average values of two measurements of eGFR taken over a 3-month interval were used

^b^Comparison between RA patients with renal dysfunction and those without

### Current use of RA medications in RA patients with and without renal dysfunction

In terms of RA medications, 51.6% of RA patients were receiving MTX, and 33.6% were currently being treated with biological DMARDs (Table 3). NSAIDs and steroids were used in 25.6 and 30.7% of patients, respectively. The use of MTX was significantly less frequent in RA patients with renal dysfunction defined according to absolute eGFR values (43.6 versus 55.7%, *p* < 0.001) and in those categorized with BSA-indexed eGFR (39.4 versus 54.3%, *p* < 0.001) (Tables 3 and 4). The mean weekly MTX dose in users was also significantly lower in the group with absolute eGFR-based renal dysfunction (6.2 versus 8.2 mg/week, *p* < 0.001) and in the group with BSA-indexed eGFR-based renal dysfunction (6.7 versus 8.4 mg/week, *p* < 0.001), but there was no significant difference in median cumulative MTX doses in users between the renal dysfunction group and the group without this complication. Biological agents were used more often in the group with absolute eGFR-based renal dysfunction (37.1 versus 31.8%, *p* = 0.024). Steroid user rates were significantly higher in the group with BSA-indexed eGFR-based renal dysfunction (35.8 versus 29.5%, *p* = 0.022). Daily steroid doses in users were similar between the two groups, but the median cumulative dose in users was significantly higher in the absolute eGFR-based renal dysfunction group (5.0 versus 3.1 g, *p* < 0.001) and in the BAS-indexed eGFR-based renal dysfunction group (5.0 versus 3.2 g, *p* < 0.001). There was no significant difference in rates of prescription of NSAIDs between the two groups in either the use of absolute eGFR or BAS-indexed eGFR for the definition of renal dysfunction. In addition, there were no significant differences in ASAS-NSAID scores or rates of concurrent steroid use with NSAIDs between both groups.

### Multivariate logistic regression analyses

Results from multivariate logistic regression analyses are shown in Table 5. Age (≥ 65 years), female sex, RA duration (≤ 3 years), RA stages III and IV, HAQ (≥ 1.0), serum CRP levels, BMI (> 25), hypertension, NIDDM, serum LDL-C (≥ 140 mg/dl), smoking history, biological DMARD use, NSAID use, and cumulative steroid dose (≥ 5.5 g) were selected as independent variables. We did not included MTX use or weekly MTX dose as a possible risk factor in analyses because the negative association seen in the univariate analyses was explained by the therapeutic avoidance of MTX by the treating rheumatologists. Regarding absolute eGFR-based renal dysfunction, advanced age (OR 7.24, *p* < 0.001), female sex (OR 3.12, *p* < 0.001), and hypertension (OR 2.22, *p* < 0.001) were the strong factors independently and positively associated with renal dysfunction in RA patients. Obesity (OR 0.59, *p* < 0.001) was the strong factor independently and negatively associated with renal dysfunction. High serum LDL-C levels, NSAID use, and high cumulative doses of steroid remained in the final model, but their associations were marginal.

**Table 5:** Factors associated with renal dysfunction in RA patients

	OR	95% CI	*p* value
Absolute eGFR-based renal dysfunction			
Age (≥ 65 years)	7.24	5.71–9.18	< 0.001
Female sex	3.12	2.29–4.24	< 0.001
BMI (> 25)	0.59	0.44–0.78	< 0.001
Hypertension	2.22	1.76–2.80	< 0.001
Serum LDL-C (≥ 140 mg/dl)	1.38	1.01–1.89	0.046
NSAID use	0.77	0.60–1.00	0.050
Cumulative steroid dose (≥ 5.5 g)	1.40	1.00–1.95	0.049
BSA-indexed eGFR-based renal dysfunction			
Age (≥ 65 years)	5.19	3.83–7.05	< 0.001
Female sex	1.38	0.99–1.92	0.061
RA duration (≤ 3 years)	0.72	0.51–1.01	0.058
Serum CRP levels	1.12	0.99–1.27	0.068
Hypertension	3.05	2.37–3.96	< 0.001
NIDDM	1.52	1.06–2.18	0.022
Serum LDL-C (≥ 140 mg/dl)	1.53	1.08–2.17	0.016
Cumulative steroid dose (≥ 5.5 g)	1.45	1.01–2.08	0.047

Two separate multivariate logistic regression analyses were conducted to evaluate factors associated with the risk of renal dysfunction based on absolute eGFR and BSA-indexed eGFR. Independent factors that remained in the final models are shown. The final step yielded an AUC-ROC of 0.79 (95% CI 0.77–0.81, *p* < 0.001) for absolute eGFR-based renal dysfunction and 0.78 (95% CI 0.76–0.81, *p* < 0.001) for BSA-indexed eGFR-based renal dysfunction

*RA* rheumatoid arthritis, *eGFR* estimated glomerular filtration rate, *BMI* body mass index, *LDL-C* low-density lipoprotein cholesterol, *NSAID* nonsteroidal anti-inflammatory drug, *BSA* body surface area, *CRP* C-reactive protein, *NIDDM* non-insulin-dependent diabetes mellitus, *OR* odds ratio, *95% CI* 95% confidence interval, *AUC* area under the curve, *ROC* receiver operating characteristics

Independent and strong associations of BSA-indexed eGFR-based renal dysfunction with advanced age (OR 5.19, *p* < 0.001) and hypertension (OR 3.05, *p* < 0.001) were confirmed (Table 5). Diabetes mellitus (OR 1.52, *p* = 0.022) and high serum LDL-C levels (OR 1.53, *p* = 0.016) were also significant factors associated with renal dysfunction but their associations were less potent after adjustment for other covariates. The association between renal dysfunction and high cumulative steroid doses was marginal. Female sex, RA duration, and serum CRP levels remained in the final model, but their associations were not significant.

## Discussion

In the present study, the prevalence of renal dysfunction defined as absolute eGFR < 60 ml/min was 33.8% in RA patients, whereas 18.6% had renal dysfunction defined as BSA-indexed eGFR < 60 ml/min/1.73 m^2^. Albuminuria and hematuria were observed in 8.1 and 7.5% of RA patients in our cohort, respectively. In multivariate regression analyses, advanced age and hypertension were found to be the strong factors independently associated with the risk of renal dysfunction when it was defined using either absolute eGFR or BSA-indexed eGFR values. The strong association of female sex (positive association) and obesity (negative association) was observed only with absolute eGFR-based renal dysfunction. Diabetes mellitus, high serum LDL-C levels, and high cumulative steroid doses were significantly associated with renal dysfunction, but these associations were less potent than advanced age and hypertension.

Few studies have determined the prevalence of renal dysfunction defined according to eGFR data in RA patients. A recent study using a nationwide database of rheumatic diseases in Japan (the NinJa study) showed that the prevalence of BSA-indexed eGFR ≥ 30 and < 60 ml/min/1.73 m^2^, ≥ 15 and < 30 ml/min/1.73 m^2^, and < 15 ml/min/1.73 m^2^ was 17.5, 0.8, and 0.2%, respectively, which was very similar to the data obtained in the present study [32]. In a nationwide, large-scale epidemiological study for the Japanese general population, Imai et al. showed that the prevalence of renal dysfunction defined as BSA-indexed eGFR < 60 ml/min/1.73 m^2^ was 20.5% [33]. This is similar to the prevalence in the RA cohort of the present study and in that of the NinJa study (18.6% in both studies). In studies from Western countries, the prevalence of renal dysfunction defined as BSA-indexed eGFR < 60 ml/min/1.73 m^2^ in RA patients was reported to be 8.8% (the UK), 12.8% (France), and 15.0% (France) [4, 5, 8]. Hickson et al. reported in a follow-up study for an inception RA cohort in the USA that the 20-year cumulative incidence of renal dysfunction was higher in RA patients than in non-RA participants (25 versus. 20%, *p* = 0.03) [7].

The aforementioned studies used BSA-indexed eGFR values in determining the prevalence of renal dysfunction; however, indexing of eGFR for BSA can have a substantial impact on clinical studies involving patients with extreme body sizes, namely, an underestimation of eGFR in the case of obese patients and an overestimation of GFR in the case of underweight patients [28, 29]. In the present study, the mean absolute eGFR value was significantly lower than that of BSA-indexed eGFR values (69.0 ml/min versus 78.4 ml/min/1.73 m^2^), and the rate of patients with absolute eGFR-based renal dysfunction was nearly twofold higher than the rate of patients with renal dysfunction determined based on BSA-indexed eGFR values (33.8 versus 18.6%). These results can be explained by the fact that a considerable number of participants had extremely low BSA values (mean, 1.52 m^2^). Considering that RA patients, especially elderly individuals, are likely to present a smaller body size than 1.73 m^2^, it would be better to consider using absolute eGFR instead of BSA-indexed eGFR in studies for RA patients. The Chronic Kidney Disease Epidemiology Collaboration (CKD-EPI) equation and the Modification of Diet in Renal Disease (MDRD) equation are currently the most widely used methods for the estimation of BSA-indexed eGFR in clinical practice as well as clinical studies. Recently, Redal-Baigorri et al. showed that the performance of these formulas could be significantly improved in the assessment of individual kidney function if absolute values are used by removing the BSA normalization factor. In that study, absolute eGFR by CKD-EPI is comparable to measured GFR [30].

Over the last few years, the causative role of inflammation in the development of renal dysfunction and disease has been the subject of controversy. Two prospective follow-up studies on patients with inflammatory rheumatic diseases showed that the severity of psoriasis and the presence of psoriatic arthritis were significant factors associated with CKD [34, 35]. Regarding RA patients, in a 5-year follow-up study, Chiu et al. showed that, after adjusting for traditional cardiovascular risk factors, RA patients still had a higher risk of developing CKD compared with individuals without RA, and they suggested that chronic inflammation may be one of the main factors that contributed to the increased CKD risk in RA patients [17]. In a retrospective observational study, Kochi et al. showed that, even after adjusting for classical CKD risk factors, a persistently high level of CRP remained a significant risk factor for the development of CKD in RA patients [18]. In contrast, Daoussis et al. showed that renal dysfunction is not related to RA-related factors, with the exception of the presence of extra-articular complications, including disease activity and duration, disability, and the use of RA medications [5]. Couderc et al. also revealed that neither disease activity nor severity of RA is associated with renal impairment [8]. Haroon et al. indicated that, in patients with RA or seronegative inflammatory arthritis, no association was noted between renal dysfunction and the use of inflammatory markers, duration of arthritis, or DMARDs [16].

In the present study, traditional cardiovascular risk factors, such as advanced age and hypertension, were identified as the factors independently associated with renal dysfunction in RA patients. Several studies also identified some traditional cardiovascular risk factors as being independently associated with the risk of developing renal dysfunction in RA patients [5, 7, 8, 16, 17]. Considering recent advances in the control of RA disease activity and the decreased frequency of nephrotoxic DMARD use, the impact of RA-related factors may have become limited. Instead, it may be traditional cardiovascular risk factors that mainly contribute to the development of renal damage in RA patients. The prevalence of traditional risk factors is apparently increasing in the general population, which may be the cause of the increased prevalence of decreased renal function [36]. This seems to be true for RA patients.

NSAIDs have been considered to cause renal damage. Chiu et al. showed that NSAIDs are associated with an increased risk of CKD in RA patients [17]. In the present study, however, the use of NSAIDs was not identified as a factor associated with renal dysfunction in RA patients, and there was no significant difference in ASAS-NSAID scores between the groups with and without renal dysfunction. Similarly, Couderc et al. revealed that there was no significant association between renal dysfunction and the use of NSAIDs in RA patients. ASAS-NSAID scores did not differ between the two groups [8]. Moller et al. recently showed in a long-term prospective study that chronic use of NSAIDs had no negative impact on renal function estimates for RA patients with moderate renal dysfunction, defined as eGFR < 60 and ≥ 30 ml/min [37]. Several studies indicated that NSAID use was significantly associated with a higher GFR compared with nonusers and suggested that NSAIDs may have been prescribed or taken by patients with better renal function at baseline [5, 7, 16].

Our findings are subject to at least two limitations. First, the design of the present study was cross-sectional, which cannot provide any indication as to the sequence of events. Even when strong statistical evidence indicates that a variable is an independent risk factor for an outcome, this does not necessarily indicate that the risk factor causally contributes to the outcome [38]. Therefore, it was difficult to make a causal inference. Second, we used the eGFR estimating equations instead of direct measurements of GFR using a clearance of exogenous filtration markers. Muscle wasting is a common feature in RA patients, and since serum creatinine levels are influenced by muscle mass, eGFR values calculated by using serum creatinine levels may overestimate renal function in RA patients. In clinical practice, however, the direct measurements of GFR are impractical because they are expensive and time consuming.

In conclusion, renal dysfunction is common in RA patients. When determined according to BSA-indexed eGFR, its prevalence is comparable with that in the general population of Japan. However, the use of absolute eGFR may be a better choice for the evaluation of the renal function of RA patients. Advanced age and hypertension are the strong and independent factors associated with renal dysfunction in this patient population, while the association of RA-related factors with renal dysfunction appears to be less potent. This may be explained by recent advances in the management and control of systemic inflammation associated with RA. Regular monitoring of renal function should be implemented, especially for elderly RA patients with hypertension. Identification of the high-risk group for renal dysfunction can not only facilitate early intervention to prevent this complication but can also help to achieve optimal management of RA patients.
